# An evolving paradigm of cancer stem cell hierarchies: therapeutic implications

**DOI:** 10.7150/thno.41647

**Published:** 2020-02-10

**Authors:** Alexander J Cole, Adetunji P Fayomi, Vivian I Anyaeche, Shoumei Bai, Ronald J Buckanovich

**Affiliations:** 1 Department of Internal Medicine and Magee-Womens Research Institute, University of Pittsburgh, Pittsburgh, PA, USA.; 2 University of Pittsburgh, School of Medicine, Pittsburgh, PA, USA.; 3 Division of Gynecologic Oncology, Department of Obstetrics and Gynecology, Hillman Cancer Center, University of Pittsburgh, Pittsburgh, PA, USA

## Abstract

Over a decade of research has confirmed the critical role of cancer stem-like cells (CSCs) in tumor initiation, chemoresistance, and metastasis. Increasingly, CSC hierarchies have begun to be defined with some recurring themes. This includes evidence that these hierarchies are 'flexible,' with both cell state transitions and dedifferentiation events possible. These findings pose therapeutic hurdles and opportunities. Here, we review cancer stem cell hierarchies and their interactions with the tumor microenvironment. We also discuss the current therapeutic approaches designed to target CSC hierarchies and initial clinical trial results for CSC targeting agents. While cancer stem cell targeted therapies are still in their infancy, we are beginning to see encouraging results that suggest a positive outlook for CSC-targeting approaches.

## Introduction

The concept of adult stem cells initially evolved from landmark studies in the 50's and 60's which demonstrated the ability of transplanted bone marrow cells to rescue irradiated mice by restoring normal blood pathology 1,2. These cells were later termed hematopoietic stem cells (HSCs) and demonstrated to exist in an undifferentiated quiescent state at the peak of a differentiation hierarchy. When stimulated to proliferate, HSCs were shown to yield two distinct cells; one non-dividing (quiescent) stem cell and one actively dividing cell. This phenomenon was termed “asymmetric division”. The proliferating daughter cell was shown to continue to divide and proceed down the hematopoietic hierarchy, from stem cell to progenitor cell, before becoming a fully differentiated mature blood cell. Thus, stem cells, since, have been defined by their ability to self-renew and give rise to a well-differentiated progeny 3. Since these initial studies, multiple types of stem cells have been identified in a wide range of tissue sharing the multipotency characteristics of HSCs.

The first studies suggesting cancer cells may share similar stem cell properties to HSCs were conducted in teratomas, where it was demonstrated that undifferentiated cells preferably gave rise to non-tumorigenic differentiated cells 4. This led researchers to propose the first cancer stem cell hypothesis, that tumors comprise a mixture of malignant stem cells and their benign progeny 5. Shorty following this, a population of leukemia stem cells, which could initiate leukemia in mice, was identified 6. CSCs, defined as cells which can undergo asymmetric division and initiate tumors in mice, have now been identified in a wide variety of tumor types, including melanoma, osteosarcoma, leukemia, breast, colorectal, brain, prostate, pancreatic, ovarian, liver and lung 7. In some cancers, it has not been possible to distinguish CSCs from non-CSCs 8. Such tumors may have a very shallow hierarchy, or a differentiation block at the level of the CSC 8.

In addition to the ability to self-renew and differentiate, CSCs share a number of unique features which set them apart from bulk tumor cells. Epithelial CSCs express many genes/pathways typically associated with normal stem cells, such as SOX2 9, NANOG 10, OCT3/4 11, and the WNT/ß-Catenin 12 and Hedgehog pathways 13. In many tumor types, CSCs, or a subset of CSCs, take on an epithelial-to-mesenchymal transition (EMT) profile through the upregulation of genes such as TWIST, SNAIL, and ZEB 14,15. It is therefore unsurprising that CSCs have been demonstrated to drive metastasis in a number of cancer types 16,17. One of the more controversial features of CSCs is innate chemoresistance. While innate chemoresistance is not required to define a CSC, innate therapy resistance has been commonly linked to CSCs. This resistance has been attributed to the ability to become quiescent 18, upregulation of enzymes (such as ALDH) and multidrug resistance pumps to increase chemotherapy elimination from the cell 19, and the upregulation of anti-apoptotic proteins 20. Given their link with tumor initiation and drug resistance, they have been pushed to the forefront of cancer therapy.

The identification of CSCs is based on expression of a variety of cell surface makers, enzyme activity, transcription factors, and efflux pumps. Some are tissue specific, while others relate to pathways known to be essential for the function of normal stem cells. For a summary of these markers, we refer the reader to the review article 21. Here, we will focus our review on the differentiation capacities of CSC populations.

## CSC hierarchies

**Figure 1:**
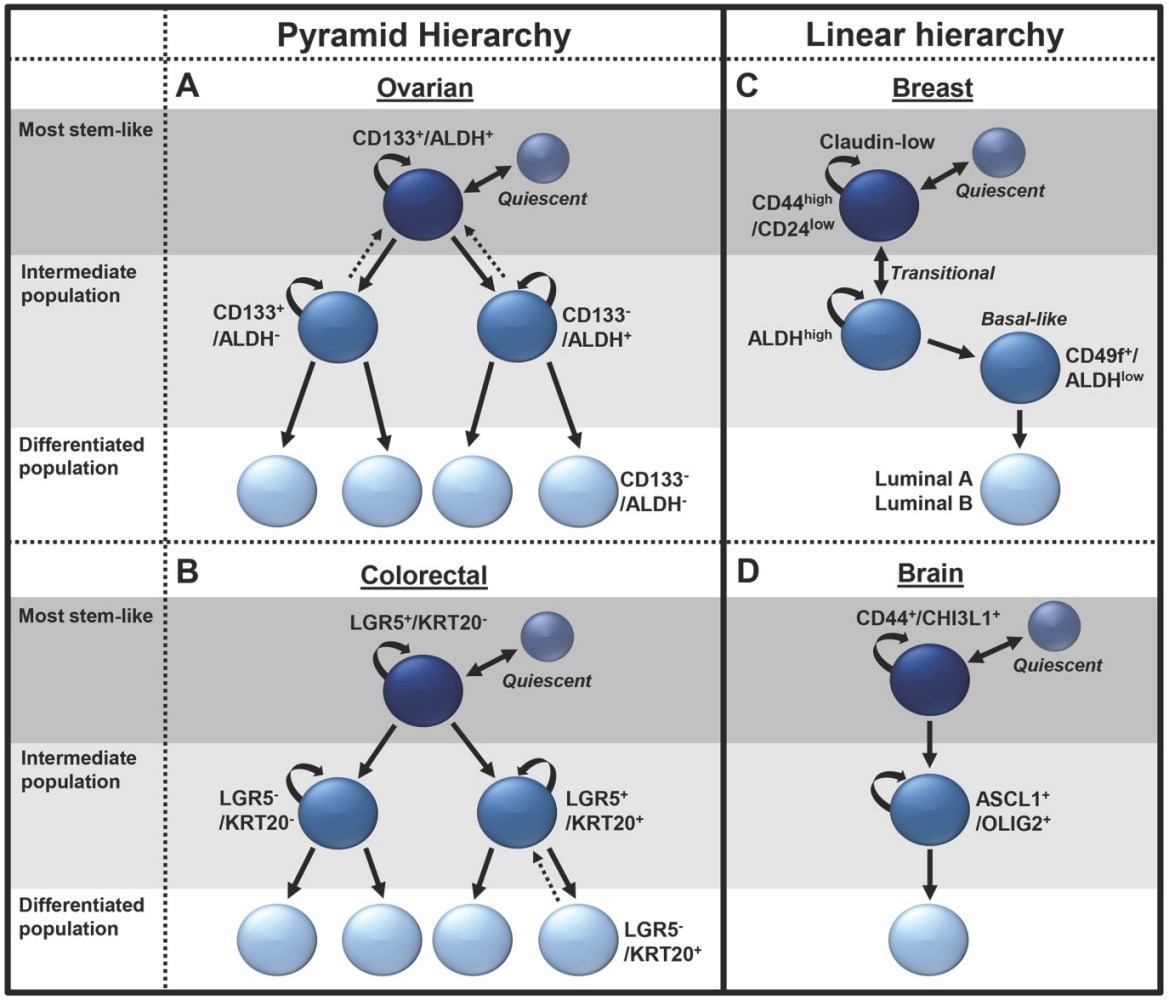
** Examples of pyramid and linear CSC hierarchies.** Unbroken arrows represent differentiation events. Broken arrows represent dedifferentiation events. Two-way arrows represent transitional differentiation events.

The CSC hypothesis postulates that many heterogenic cancers are organized into hierarchal structures based on differentiation capacity, similarly to HSC organization. The top tier of these CSC hierarchies generally contains the most stem-like cells, capable of self-renewal and differentiation into the less stem-like cells which comprise the lower tiers of the hierarchy (**Figure 1**). These apex CSCs typically have prodigious tumor initiation capacity and are responsible for driving tumor heterogeneity and composition of the bulk tumor mass and facilitating tumor growth, drug resistance, cancer recurrence, and metastasis. The differentiation hierarchy model of cancer is dependent on the gain and loss of the various markers used to identify the specific CSC populations. As noted above, tissue-specific gene expression results in a wide variety of CSC markers being identified for many different types of cancer and multiple markers for the same cancer type. Consequently, the literature reflects a conglomerate of CSC types, markers, and models. Recently, studies have begun to identify and characterize CSC hierarchies in several cancer types, including ovarian 22, colon 23, breast 24, and brain cancers 25. With the advances in lineage tracing and single-cell sequencing technologies, it is likely CSC hierarchies will be defined in other tumor types. We believe that understanding these hierarchies will identify critical therapeutic targets to improve patient outcomes. Below, we will highlight some defined CSC hierarchies and the role of quiescent CSCs within the hierarchy. We will also discuss dedifferentiation and epigenetic alterations as a source of 'stemness' and the impact of the tumor microenvironment (TME) on CSCs.

### Example stem cell hierarchies

Ovarian cancer: Using the previously identified ovarian CSC markers ALDH and CD133 26,27, Choi, et al., (2015) defined an ovarian cancer CSC differentiation hierarchy (**Figure 1A**) 22. This study used single cell-lineage tracing of cell lines and primary human ovarian cancer samples, defining a branched differentiation hierarchy with at least four distinct ovarian cancer cell populations. ALDH^+^/CD133^+^ cells sit at the apex of the hierarchy and symmetrically divide to expand CSC numbers in a process enhanced by bone morphogenetic protein 2 (BMP2) secretion from lower-tier cells 21. Alternatively, ALDH^+^/CD133^+^ cells can asymmetrically divide to self-renew and produce either ALDH^+^/CD133^-^ or ALDH^-^/CD133^+^ cells 22. These ALDH^+^/CD133^-^ and ALDH^-^/CD133^+^ cells comprise an intermediate CSC population, possessing enhanced tumorigenic potential over the bulk cells, but are less stem-like than the ALDH^+^/CD133^+^ cells. ALDH^+^/CD133^-^ and ALDH^-^/CD133^+^ also have the capacity for symmetric division, to expand, or asymmetric division, producing ALDH^-^/CD133^-^ cells. Interestingly, the ALDH^+^/CD133^-^ and ALDH^-^/CD133^+^ cells each have distinct features. ALDH^+^ cells are known to preferentially grow in suspension/spheroids and to demonstrate platinum resistance, with ALDH expression regulated by β-catenin 26. In contrast, CD133^+^ cells are slower growing 22,28 and appear more radiation resistant 29. The bottom tier of the stem cell hierarchy comprise ALDH^-^/CD133^-^ cells, which make up the majority of the ovarian cancer mass and are generally unable to initiate tumors 26. The lack of stemness in ALDH^-^/CD133^-^ cells prevent their growth as spheroids and make them sensitive to chemotherapeutics 22,26. One study suggested CD133^-^ cells could initiate tumors. However, this study, like many, is difficult to interpret, as large numbers of cells were used; thus, FACs contamination is problematic 30. However, single-cell studies did observe rare dedifferentiation events wherein ALDH^-^/CD133^-^ cells could gain expression of CSC markers 22.

Support for this hierarchy also comes from ovarian cancer cell-of-origin studies in mice, where a lineage-tracing study suggested LGR5^+^, ALDH^+^/CD133^+^ ovarian surface-repopulating stem cells were found in the oviductal hila of mice. When mutated, these cells could serve as CSCs 31. This ovarian CSC hierarchy is an example of a classic pyramid of differentiation.

Colorectal cancer: Like ovarian cancer, colorectal cancer has a branched CSC differentiation hierarchy **(Figure 1B)**. This hierarchy recapitulates normal stem cell organization in the colon 23,32,33. Using lineage-tracing in organoids Shimokawa et al., (2017) defined a colorectal CSC hierarchy based on the expression of the CSC marker LGR5 and the differentiation marker KRT20. In this hierarchy, the apex cells LGR5^+^/KRT20^-^ can self-renew or differentiate into LGR5^+^/KRT20^+^ or LGR5^-^/KRT20^+^ cells 23. At the bottom of the hierarchy, the LGR5^-^/KRT20^+^ exist as terminally differentiated, non-proliferative cells. Similar to the findings in ovarian cancer, in rare incidences, LGR5^-^/KRT20^+^ cells could revert into LGR5^+^/KRT20^+^, form colonies and proliferate. Interestingly, transplantation of the colorectal organoids *in vivo* resulted in a recapitulation of the original cancer tissue histology, with the stem-like LGR5 cells localized to the outermost regions of the tumors, surrounded by α-smooth muscle actin-positive fibroblasts and KRT20^+^ differentiated cells localized to the inner regions 32,33.

Breast cancer: The breast CSC hierarchy has been largely inferred from the well-defined normal breast stem cell hierarchy, with the apex cell proposed as a source of claudin-low/triple-negative breast cancers, bipotent and luminal progenitors driving basal like tumors, and ductal progenitors leading to ER^+^ luminal A/B tumors 34. While the normal breast stem cell hierarchy is clearly branched, it is not clear that all branches are associated with breast cancer, thus we propose a more linear CSC hierarchy. The top tier of the breast CSC hierarchy is composed of CD24^low^/CD44^high^ EMT/mesenchymal-like cells, which differentiate into an intermediate stem cell population made up of ALDH^high^ epithelial-like cells (**Figure 1C**) 24. The cells in the mesenchymal state are slower growing and localized to the invasive front of the tumor, while the epithelial-like cells are more rapidly growing and typically located in the center of the tumor mass 24. Making it distinct from the other branched models, the breast CSC model has significantly more plasticity, allowing rapid cell state transitions between the CD24^low^/CD44^high^ and ALDH^high^ stem cell populations. Recent evidence has also suggested a second intermediate breast basal-like CSC population comprising ALDH^low^/CD49f^+^ cells, which are less stem-like than ALDH^high^ cells 35. The lowest tier of the hierarchy comprises luminal A/B type cells, which are the most well differentiated and the least stem-like. The enhanced plasticity of this hierarchy and the ability of these cells to transition between the mesenchymal and epithelial states may help them respond to environmental stress. Indeed, the phenotypes for these states may also be more plastic than initially realized, as more recent research has contradicted the roles of these cells, suggesting the CD24^low^/CD44^high^ population to be the most proliferative and tumorigenic, while the ALDH cells were identified to be more migratory and to promote tumor metastasis 36.

Brain cancer: Recent advances in single-cell RNA sequencing have overcome the technical barriers for lineage tracing and rendered higher resolution of cancer biology possible 37. Patel, et al., (2014) sequenced 430 cells from five primary glioblastomas. Analysis confirmed subtype classification and revealed significant tumor heterogeneity 38. This study further identified a population of CD133^+^ quiescent glioblastoma stem cells that were enriched for hypoxia signatures 38. Recently, Wang, et al., (2019) further delineated proliferating glioblastoma CSCs into a linear differentiation hierarchy (**Figure 1D**). In this model, parallel to the breast cancer model, a mesenchymal CSC population, expressing the glycoprotein markers CD44 and CHI3L1, was the most stem like. These cells were demonstrated to differentiate into pro-neural cells expressing the transcription factors ASCL1 and OLIG2. These intermediate pro-neural progenitors in turn differentiated into a bulk cell population 39.

## Quiescent subpopulations

Early CSC studies focused on tumor initiation capacity in animals, and thus by design typically identified rapidly growing CSC populations. However, recent work is beginning to show a critical role for quiescent CSCs in cancer biology and, specifically, in therapeutic resistance. Quiescence describes a reversible state of cellular inactivity, in which a cell has exited the cell cycle into the G0 phase, where it will remain until reentering the cell cycle in response to physiological cell stimuli. Quiescent cells are typically resistant to chemotherapies that target proliferative cells.

Quiescent CSCs likely make up a sub-population of CSCs with enhanced resilience to environmental stresses 18,40. Quiescent CSCs, defined by various markers, have been reported in breast 24, liver 41, melanoma 42, ovarian 40, colon 43, and brain CSCs 38. Importantly, numerous studies across cancer types demonstrate quiescent CSC contribute to chemotherapy resistance and tumor recurrence 44. For example, studies in pancreatic cancer identified a slow-cycling population, enriched for the CSC markers CD24^+^/CD44^+^, CD133^+^ and ALDH, which also had enhanced chemotherapeutic resistance and could recreate the initial heterogeneous tumor cell population 18. Several studies link expression of SOX2 with quiescent CSCs 45,46. We investigated the role of the nuclear factor of activated T-cells (NFAT) family, transcription factors known to regulate quiescence in normal stem cells 28, as regulators of quiescent ovarian CSCs. We found that NFATC4 is enriched in slow proliferating ovarian CSCs and is increased in response to cisplatin therapy, while overexpression of NFATC4 was shown to cause a marked decrease in proliferation and cell size, G0 cell cycle arrest, and chemotherapy resistance 28. Understanding quiescent CSCs thus offers an important opportunity to overcome therapeutic resistance to prevent disease recurrence. Alternatively, the ability to force a quiescent state in residual cancer cells post-therapy would also be of interest to prolong patient progression-free survival. Consistent with this, Chesnokov, et al., (2019) demonstrated that MEK inhibitors induce a G0/G1 cellular arrest with induction of stemness genes SOX2, NANOG, OCT4, and ALDH1A homologs 47.

## Plasticity of the hierarchy and dedifferentiation as a source of stemness

Consistent with findings in normal tissues, such as the lung, where cells can undergo lineage switching/dedifferentiation in the face of injury 48, CSC hierarchies are more flexible than originally hypothesized, with significant cellular plasticity. Since initial studies identifying dedifferentiation events of bulk breast cancer cells to breast CSCs both *in vitro* and *in vivo*, CSC hierarchies have evolved to incorporate *de novo* generation of CSCs from what was once believed to be terminally differentiated cells 49. This bidirectional interconversion of non-CSCs to CSCs gives hierarchies the ability to respond to cellular stresses by alternating their differentiation from a mesenchymal to an epithelial phenotype, or de-differentiating up the hierarchy from non-stem-like to stem-like cells to recover an ablated population 23,24. This plasticity poses a therapeutic challenge, but also indicates the critical importance of the stemness. In addition, it offers a potential therapeutic opportunity, as blocking the induction of a state of stemness could represent a means to restrict cancer growth.

The phenomenon of CSC dedifferentiation has been explored in many different tissue models including breast 50, lung 51, melanoma 52, ovarian 22, glioma 53, pancreas 54, and colon 55. However, dedifferentiation studies are a challenge, as experiments using bulk cells often cannot rule out trace contamination of hard-to-detect or previously undefined CSC pools. As such, dedifferentiation studies need to be viewed under a critical lens, with single-cell studies representing the ideal.

Various general mechanisms of dedifferentiation appear to be emerging. One mechanism is EMT driven, where TGF-β or other factors activate the EMT-associated transcription factors (TWIST, SNAIL/SLUG, or ZEB) to assume a stem-like state 50,55-57. However, this may be more a state transition than a true dedifferentiation phenomenon. An alternative mechanism is via induction of core stem cell, 'Yamanaka,' transcription factors, essential for the reprogramming of somatic cells into induced pluripotent stem cells. These factors include; OCT, SOX, MYC and KLF family members, in addition to NANOG and Lin-28. In melanoma, lung, pancreatic, and colon cancers, OCT4 was demonstrated to promote dedifferentiation of bulk cells into CSC 51,52,54,58, while upregulation of NANOG, SOX2, Klf and Lin-28B have also been implicated in this process 51,54,58. Studies in iPS cells suggest that the EMT transcription factors may prime cells for Yamanaka factor induced cellular reprogramming 59. Similar mechanisms may take place in cancer cells 60.

The capacity of CSCs to dedifferentiate under stressful conditions, such as hypoxia, radiation, or stem cell ablation, has also been widely reported 23,61,62, and seems to favor the Yamanaka transcription factor pathways 62-64. In contrast, dedifferentiation events caused by the treatment of bulk cancer cells with chemotherapy seem to utilize not only the SOX2 and OCT3/4 pathways 65, but also stemness genes and CSC markers such as notch and ALDH1 66. Interestingly, the reactivation of many of these stemness genes have been associated with epigenetic changes such as promoter hypomethylation, and will be discussed below. Various other pathways have also been implicated in dedifferentiation, including; cell cycle activators and developmental genes; however, these mechanism seem to be less common 53.

A new source of dedifferentiation comes from the formation of polyploid giant cancer cells (PGCCs). These cells express the stemness genes OCT4, NANOG and SOX2/4 67 and have been demonstrated to asymmetrically differentiate to produce cells with increased tumor initiation, immunosuppressive properties, decreased sensitivity to chemotherapeutics and enhanced stemness 68,69. In a study by Zhang, et al., (2014), tumors formed from PGCCs were shown to possess a mesenchymal phenotype and have elevated expression of the CSC markers CD44 and CD133 69. Interestingly, PGCCs tend to cycle slowly and are thus a potentially important source of quiescent CSC 69.

## Epigenetics role in CSCs

Epigenetics is a broad term used to encompass a wide range of mechanisms capable of altering gene expression without altering the DNA sequence. These mechanisms include DNA methylation, histone modifications, noncoding RNAs, and chromatin remodeling. Epigenetics plays a pivotal role in the normal function of embryonic and adult stem cells, controlling their ability to differentiate, self-renew, and maintain pluripotency 70,71. CSCs also rely on a range of epigenetic modulators to maintain and promote their stemness programming. Numerous studies have suggested CSCs possess altered epigenetic landscapes compared with bulk tumor cells 72-74. The CSC marker CD133 has been shown to be hyper-methylated in bulk cells compared to the CSC population 73,74, while CD133 and CD44 were shown to be hypomethylated and subsequently overexpressed in triple-negative breast cancer compared to non-triple-negative 72. In addition to direct effects on CSC markers, epigenetics has been shown to regulate stemness pathways, such as Wnt/β-catenin 75, hedgehog, notch and TGF-β. Recent work by Wang, et al., (2018) demonstrated that the imprint gene ASCL2 is required to maintain Wnt activation in CSCs. In CSCs, ASCL2 is epigenetically regulated by the histone methyltransferase SMYD3, which regulates H3K4me3 status at the ASCL2 locus, promoting it's expression 76. The non-coding RNA lncTCF7 has also been shown to promote Wnt signaling via recruitment of the chromatin-remodeling complex SWI/SNF 77. Hedgehog signaling is another target for epigenetic regulation by CSC. A recent study by Ooki, et al., (2018) demonstrated that in bulk lung cancer populations the promotor of the transcription factor PAX6 was methylated, resulting in its repression, while in CSCs PAX6 was not methylated and promoted transcription of the hedgehog regulator GLI, resulting in an upregulation of SOX2, OCT4, and NANOG, driving cancer cells toward a stem-like state 78. Jin, et al., (2017) confirmed the ability of CSCs to epigenetically regulate notch, demonstrating CSC-specific upregulation of STRAP, which disrupted polycomb repressor complex 2 assembly, preventing the silencing of notch and promoting a stem-like phenotype 79. Several studies have also demonstrated the ability of microRNAs to promote stemness. miR-200c and miR-205, responsible for silencing ZEB1, can be epigenetically silenced, resulting in an upregulation of EMT and CSC phenotypes 80,81.

## The impact of the tumor micro-environment on stemness

**Figure 2:**
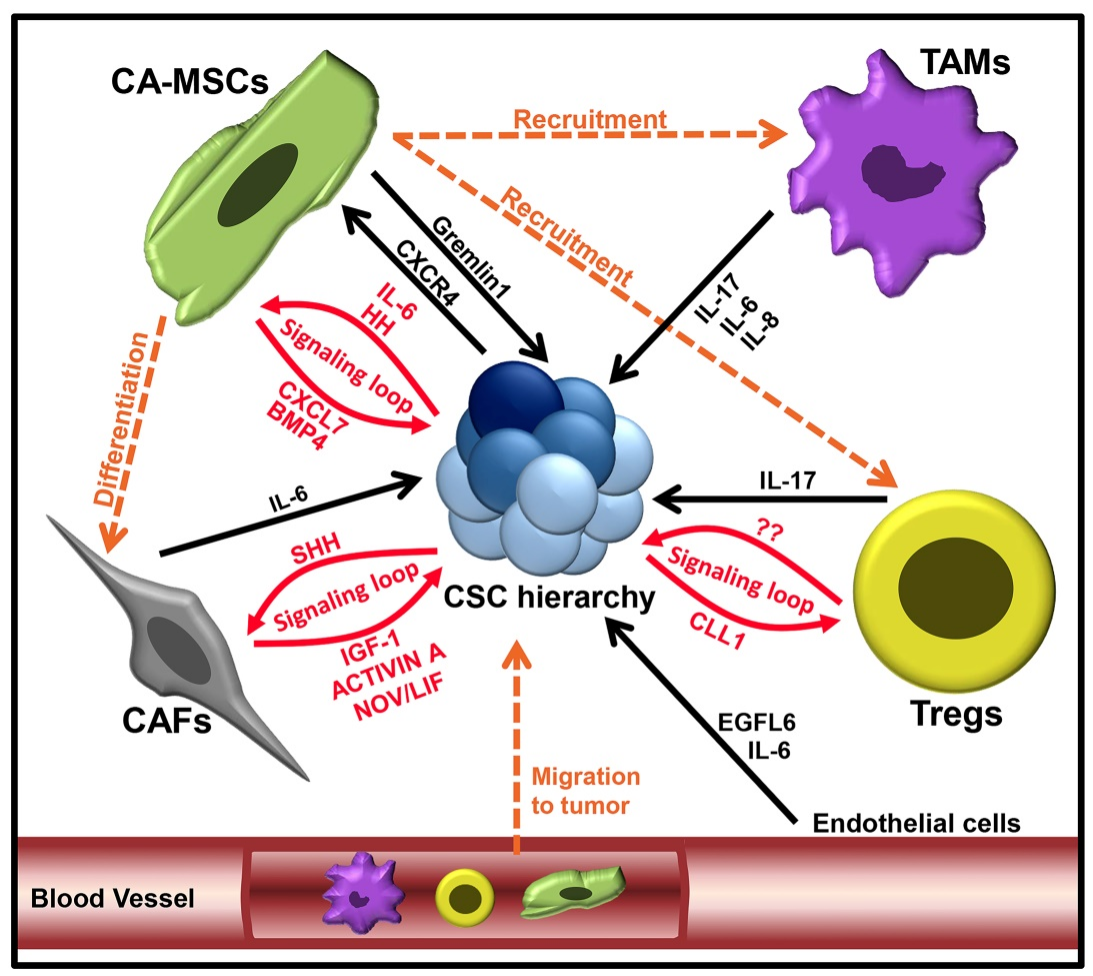
Tumor microenvironment factors which regulate the CSC hierarchy expansion.

Over the past decade, cancer research has shifted away from solely targeting the bulk tumor mass, to focusing on the TME. Cells of the TME have been demonstrated to be vital for the regulation of CSC hierarchies. We will focus here on the roles of mesenchymal stem cells (MSCs), cancer-associated fibroblasts (CAFs), tumor associated macrophages (TAMs), suppressive regulatory T cells (Tregs) and vascular cells in promoting cancer stemness **(Figure 2)**.

Mesenchymal stem cells are multipotent cells, able to self-renew and differentiate into various cell types, including adipocytes, chondrocytes and osteoblasts 82. In various cancer types, carcinoma associated MSCs (CA-MSCs) have been shown to promote tumor 'stemness', increasing tumor growth, chemotherapy resistance, and metastasis 83,84. One important theme appears to be the establishment of signaling loops between CA-MSCs and CSCs. In ovarian cancer, a BMP4:Hedgehog CA-MSC:CSC signaling loop was found to increase stemness and drive therapeutic resistance 84-86. In breast cancer, an MSC:CSC CXCL7:IL-6 signaling loop increased breast CSC populations, resulting in an overall increase in tumor growth 87. CA-MSCs were also shown to inhibit FOXP2 in breast CSCs, promoting tumor initiation and metastasis 88. Breast CA-MSCs have also been demonstrated to promote breast cancer quiescence and drug resistance 89. In colorectal cancer, CA-MSCs have been shown to promote the dedifferentiation of LGR5^-^ cells through production of Gremlin1 90. Numerus other studies support the critical link of CA-MSCs and CSCs; for a deeper examination of this area we refer readers to the review 91.

CA-MSCs can also impact CSCs indirectly via the generation of CAFs 92. CAFs are the most abundant cells in the TME and are responsible for regulating the biology of tumor cells through direct contact, paracrine signaling and extra-cellular matrix remodeling 93. A number of studies have demonstrated that CAFs can function similarly to CA-MSCs, via various secreted factors, to enhance CSC expansion and EMT, to promote tumor progression 33,94. For example, CAFs can secrete interleukin (IL)-6, which has been found to activate notch signaling in hepatocellular carcinoma cells, promoting CSC properties 95. Much like those observed for CA-MSCs, CAF:CSC interactions commonly appear to be reciprocal. In a study by Valenti, et al., (2017) CSCs were demonstrated to activate hedgehog signaling in CAFS, while CAFs responded by secreting factors that promoted expansion of CSCs 96. While most studies indicate that CAFs increase tumor stemness, some have suggested that CAF subsets could negatively regulate CSC populations 97. This may, in part, be related to the source of MSCs; although the majority of CAFs are probably derived from the tumor stroma, multiple studies have reported that a substantial number of tumor CAFs are derived from bone marrow MSCs 92,98.

Components of the immune system also have a direct influence on CSCs. Tumor-associated macrophages (TAMs) are reported to secrete many factors that impact CSCs 99,100. One critical factor produced by TAMs is IL-6. Like CAFs, TAMs can make high levels of IL-6 which has been shown to promote breast CSC self-renewal 101 and to enhance the CD44^+^ population in hepatoma cancer cells 102, and the CD44^+^/ALDH1^+^ populations in pancreatic CSCs 103. In addition to IL-6, TAMs have been demonstrated to secrete IL-8 and IL-17, which increase expansion of ALDH^+^ breast CSCs 104 and self-renewal of ovarian CD133^+^ CSCs 105, respectively. Furthermore, IL-8 has been shown enhance CSC self-renewal, sphere formation, migration and expression of stemness-related genes 106. In addition to paracrine signaling, TAMs can also influence CSCs via juxtacrine signaling 107.

Suppressive regulatory T cells, comprising T-regulatory cells (Tregs) and Th17 CD4^+^ T cells, are known to help cancer evade anti-tumor immunity. Evidence exists that CSCs both recruit and activate these regulatory cells, facilitating crosstalk to enhance CSC stemness and promote their growth. Yang, et al., (2011) demonstrated that Foxp3^+^ Tregs were capable of promoting colorectal cancer stemness through the secretion of IL-17 108. Xu, et al., (2018), demonstrated a Treg-CSC signaling loop, whereby breast CSCs secreted the chemokine CCL1, which resulted in the recruitment of Treg cells to the tumor 109. Treg cells were then demonstrated to increased ALDH activity, SOX2 expression and sphere formation in breast cancer cells via paracrine signaling; however, the signaling factors involved were not investigated. Co-engraftment of breast cancer and Tregs resulted in enhanced tumorigenesis, metastasis, and chemoresistance, confirming the supportive role of Tregs in breast cancer. Interestingly, it has also been demonstrated that CSCs can signal MSCs via CXCR4 signaling to induce Treg maturation 110.

Finally, the vascular niche and 'angiokines' can also regulate CSCs. Vascular endothelial growth factor (VEGF) is an important regulator of both vascular cells and CSCs 111. Endothelial cells impact CSCs via secretion of IL-6, promoting their migration 112. Similarly, a vascular factor, EGFL6, has been shown promote CSC asymmetric division, migration, and metastasis 113. For a more in-depth review of the vascular niche, we refer readers to the following review 114.

## Therapeutic approach to targeting CSCs

### Metabolism targeting therapies

ALDH1 inhibitors**:** Aldehyde dehydrogenase, primarily the ALDH1A family members, are among the best-supported CSC markers. Multiple preclinical studies indicate that ALDH1A1 or ALDH1A3 knockdown increases chemosensitivity/reverses chemotherapy resistance in ovarian, breast, and lung cancers and melanoma 115-119. Animal studies have confirmed the anti-cancer activity of broad spectrum ALDH inhibitors such as disulfiram and DEAB 120,121. Given the success of these broad-spectrum inhibitors, there has been an attempt to develop more selective ALDH1A family inhibitors. We have developed a pan ALDH1A family inhibitor, 673A, with the advantage being active regardless of the ALDH1A family member expressed. 673A preferentially depletes CD133^+^ ovarian CSCs, inhibits ovarian tumor initiation *in vitro* and *in vivo*, synergizes with chemotherapy against both breast and ovarian cancer cells, and increased tumor eradication in chemotherapy-resistant human-patient-derived xenograft models 29. A selective and potent ALDH1A1 inhibitor, CM37, was found to effectively increase cancer cell reactive oxygen species and DNA damage 122. Similar quinoline-based ALDH1A1 inhibitors showed efficacy in chemosensitization of ALDH1A1-expressing cancer cell lines 123. Newer, more clinically applicable ALDH inhibitors are being developed 124. Indeed, a novel cytotoxic ALDH-targeting pro-drug has shown significant activity in melanoma 125.

While none of these newer compounds have entered clinical trials, several older ALDH inhibitors have been tested in cancer. A phase IIb trial in patients with advanced lung cancer demonstrated that the addition of disulfiram to chemotherapy improved overall survival with ~10% of patients being disease-free at 3 years 126. Similarly, in a small study of high-risk breast cancer patients receiving adjuvant chemotherapy and sodium diethyl dithiocarbamic acid (the primary active metabolite of disulfiram), there was a trend toward increased overall and disease-free survival 127. However, combinatorial studies of disulfiram+copper and temozolomide in temozolomide-resistant glioblastoma showed limited activity 128-130. It is important to note that disulfiram, which was developed as and ALDH2 inhibitor, while active against ALDH1A1, has limited activity against. ALDH1A3, which is prevalent in many cancers. Thus, newer-generation ALDH inhibitors could significantly improve on the encouraging results of disulfiram in lung cancer.

Metformin: Metformin was first shown to target CSCs in breast cancer 131, with subsequent studies demonstrating its ability to target CSCs in many cancers 132-134. Currently, there are at least 14 trials, ongoing or concluded, evaluating the efficacy of metformin in cancer. Initial reports of metformin in pancreatic cancer were disappointing, as they demonstrated no impact on outcomes 135. Similarly, the addition of metformin to chemotherapy for HER2(-) metastatic breast cancer had no impact on progression-free survival (PFS) with overall survival (OS) not reported 136. However, more recent studies are more encouraging. We have completed a phase II study of metformin administered in combination with chemotherapy for non-diabetic patients with advanced stage epithelial ovarian cancer. This study found metformin was associated with a 2.5-fold reduction in ovarian CSCs, and while non-randomized, was associated with a surprisingly long median OS of 57.9 months 137. Significantly, a randomized phase II trial of metformin in combination with an epidermal growth factor receptor-tyrosine kinase inhibitor demonstrated a statistically significant improvement in both PFS and OS, with an impressive 14-month improvement in OS of lung cancer 138.

### Antibody therapies targeting CSC surface molecules

CD44: Expressed by many tumors, CD44 is a transmembrane glycoprotein and one of the most researched cell surface targets for CSC therapies 139. CD44 targeting therapies have shown efficacy in preclinical studies 140-142. CD44 antibody-nanoparticle conjugates selectively kill CSCs in head and neck squamous cell carcinoma 140. Anti-CD44 antibody has been shown to facilitate cellular uptake of doxorubicin, inducing chemo-sensitization 143. The first-in-human phase I clinical trial of an anti-CD44 monoclonal antibody (RG7356) for CD44-expressing local advanced or metastatic tumors was recently completed. This trial exhibited safety and efficacy and demonstrated some single-agent activity 144. Dose escalating of this antibody, in combination with various chemotherapies is ongoing. Other anti-CD44 therapies tested in ongoing or completed clinical trials include AMC303 (for solid tumors) and SPL108 (for ovarian epithelial cancer). A number of alternative strategies to target CD44 are being developed; among the most promising is the use of CD44 short binding peptides coupled to toxins, which have been shown to have 4-10 times stronger affinity to CD44 than do antibodies 141,145.

The extracellular domain of CD44 is susceptible to alternative splicing, resulting in the generation of multiple CD44 variant isoforms (CD44v). The expression of splice variant CD44v6 has been demonstrated to correlate with tumor progression and has been shown to be enriched in CSC populations 146. Targeting CD44v6 has advantages as CD44 is expressed by most cells; however, CD44v6 is only expressed in subpopulations of hematopoietic and epithelial cells. Consequently, efforts have been made to design and test CD44v6 target antibody therapies. However, an anti-CD44v6 antibody was tested in clinical trial but was discontinued due to significant skin toxicity 147,148. Another CD44v6 antibody, RO5429083, is undergoing dose escalating trials alone and in combination with various chemotherapies 149.

CD24: The sialoglycoprotein CD24 is expressed on the surface of cells, constitutes a prevalent CSC marker, has roles in cell signaling and has recently been demonstrated to promote tumor immune evasion 150. Preclinical studies with anti-CD24 antibody treatment has impeded tumor growth in hepatocellular carcinoma 151, colorectal and pancreatic adenocarcinoma 152, and reduced CSC populations 153. CD24 has been shown to be a novel 'don't eat me' signal protein, most abundantly expressed in metastatic ovarian cancer and triple-negative breast cancer. CD24 allows tumor cells to evade phagocytosis, by recognizing Siglec-10 on TAMs 150. Genetic ablation of CD24 in tumor cells, or antibodies targeting CD24, or Siglec-10, induces phagocytosis and obrogation of tumor growth. Anti-CD24 antibodies are in preclinical studies of various cancer types 154-156.

CD133 (prominin-1): The pentaspan transmembrane glycoprotein CD133is involved in WNT/β-catenin signaling and is capable of regulating cell differentiation 157,158. Currently there exist a number of CD133-targeting antibodies, such as CD133KDEL which consists of an anti-CD133 single-chain variable fragment (scFv) coupled to pseudomonas exotoxin A (PE38) 159-161. However, most of these agents are still at the preclinical stage. A recent phase I clinical trial by Wang, et al., (2018), treated 23 patients with advanced CD133-positive tumors, using autologous chimeric antigen receptor-modified T-cells (CART) expressing anti-CD133 scFv. Trial results demonstrated ablation of CD133-positive cells in all patients and an increase in disease stability without new metastasis occurring. Toxicities were manageable 162.

### Stemness pathway targeted therapeutics

Focal adhesion kinase (FAK) inhibitors: Regulated by OCT-3/4 and NANOG 163,164, FAK plays an important role in CSC self-renewal and tumor progression 165. There are more than 40 clinical trials evaluating the clinical safety or efficacy of FAK inhibitors. Seven studies have demonstrated drug tolerability, but efficacy data are limited. Reports from a phase I trial evaluating the safety of FAK inhibitor PF-04554878 when used in combination with pembrolizumab or gemcitabine in patients with pancreatic ductal adenocarcinoma demonstrated a targeted decrease in FAK phosphorylation in T cells; however, no partial or complete response was observed 166. A phase II trial of PF-04554878 in Merlin-stratified pleural mesothelioma patients after first-line chemotherapy 167 (NCT01870609) did not show an impact on PFS,OS, or quality of life.

Wnt/β-catenin inhibitors: Numerous clinical trials have been initiated to evaluate the safety and/or efficacy of various molecules targeting the Wnt/β-catenin pathway in cancer cells. In general, these drugs have shown limited activity as single agents 168. However, in combination with chemotherapy and other compounds, significant response rates have been reported. A phase Ib study of the Wnt inhibitor ipafricept in combination with nab-paclitaxel/gemcitabine in pancreatic cancer resulted a 34.6% partial response rate, 46.2% having stable disease, for an impressive clinical benefit rate of 80.8% 169. Unfortunately, this study was terminated by the sponsor before completion, possibly related to adverse side effects, though the trial data suggest clinical efficacy for Wnt inhibition.

Notch inhibitors: Monoclonal antibodies that can alter notch ligand-receptor binding and gamma-secretase inhibitors, which can block downstream signaling, are both in clinical development. Presently, there are more than 100 gamma-secretase inhibitors 170, and almost 50 clinical trials have been initiated to evaluate their clinical safety and efficacy. Early trial results indicate that the inhibitors are generally safe but associated with dose-limiting toxicities, predominantly of the gastrointestinal tract 171-173. There are conflicting results on the efficacy of notch inhibitors for cancer treatment, either as a single agent or in combination with other agents. While some studies have reported clinical benefits of notch inhibition, including stability of glioblastoma multiforme or glioma in patients 174, partial response and disease stabilization in patients with pancreatic ductal carcinoma 173, and tumor necrosis and shrinkage in patients with leiomyosarcoma and breast cancer 172, other studies have reported weight loss, higher incidence of skin cancer, deteriorating cognitive ability, and unimpressive clinical outcomes 175.

Hedgehog pathway inhibitors (HHi): As discussed above, the hedgehog signaling pathway is essential for maintaining a stem-like state and is therefore exploited by CSC via epigenetic regulation and microenvironment activation. Indeed, a combination of the HHi Daurismo plus low-dose HDAC inhibitor cytarabine vs. cytarabine alone resulted in a doubling of overall survival in elderly patients with acute myeloid leukemia, resulting in FDA approval 176. While HHi have shown significant efficacy in tumors with HHi-driver mutations, including basal cell carcinoma 177 and medulloblastoma 178, the impact as a CSC-modulating drug in HHi wildtype tumors is controversial. There are currently more than 70 phase I-IV clinical trials involving inhibitors of the Hedgehog signaling pathway aimed at eradicating bulk tumors and CSC populations 176. Over 80% of these trials use the inhibitors GDC-449 or LDE225, which bind to and inhibit Smoothened (SMO) activation, preventing downstream hedgehog signaling. Results from early trials have been underwhelming; although most studies show that the HHi are tolerated and significantly inhibit hedgehog signaling, many report no or little effect on CSC populations or improvement in patient outcomes 179-182. Currently, there is a phase III trial (NCT03416179) actively recruiting for Daurismo in combination with intensive or non-intensive chemotherapy.

IL-6/JAK/STAT: Of the secreted TME factors, IL-6 signaling via the JAK/STAT pathway has been a focus. Numerous preclinical studies have demonstrated the ability of IL-6 antibodies to inhibit CSC growth and sensitize them to chemotherapeutics 183,184. An anti-IL-6 receptor antibody has been clinically approved and used for the treatment of rheumatoid arthritis. This antibody is now in multiple clinical trials targeting cancer. Similarly, ruxolitinib, an inhibitor of JAK2 that has been approved for treatment of myeloproliferative neoplasms, is also in clinical trials for the study of several solid tumors. At least one trial, NRG007, has specific translational endpoints, studying CSCs as a target. In a phase I clinical trial non-small cell lung cancer (NSCLC) patients treated with the STAT3 inhibitor OPB-51602 where shown to have a better response to therapy 185. However, a more recent phase I trial of a novel STAT3 inhibitor in patients with advanced hepatocellular carcinoma was less promising 186.

IL-8**:** is an important cytokine secreted by the TME and bulk tumor cells to promote CSC expansion and stemness**.** Like IL-6, IL-8 is a target gene of STAT/JAK signaling. Recent studies have shown IL-8-neutralizing antibody or inhibition of the IL-8 receptors CXCR1/2 with the antagonist reparixin, abolishes breast CSCs following chemotherapy withdrawal 187. Currently, an IL-8 antibody is in phase I clinical trials for advanced solid tumors 188. In breast cancer, reparixin was safely combined with paclitaxel, with a 30% response rate and acceptable toxicities 189. Reparixin is currently in phase II clinical trial for breast cancer (NCT02370238).

CXCL12: is a chemokine secreted by CAFs which binds to the receptor CXCR4, expressed on CSCs, to promote metastasis 190. Consequently, several CXCR4 antagonists are now in clinical trial. A phase I trial of the CXCR4 peptide antagonist LY2510924 demonstrated a 20% stable disease rate in patients with advanced cancer. The most promising CXCR4 antagonist is BL-8040, which was recently granted orphan drug status by the FDA. BL-8040 is currently in five phase II trials for multiple cancer types. Early results have suggested BL-8040, in combination with pembrolizumab, is safe and shows a promising OS rate in metastatic melanoma, NSCLC, and bladder cancer 191.

### CAF targeted therapeutics

Fibroblast activation protein alpha (FAPα): FAPα is expressed in the CAFs of ~90% of all carcinomas. Substantial efforts have been made to target CAFs using FAPα 192-194, with disappointing single agent results 195. More recent trials have used FAPα inhibitors in combination with other agents; however, these, too, have shown limited clinical efficacy 196,197. Currently, there are three ongoing clinical trials (NCT03875079, NCT03386721, NCT02627274) evaluating the efficacy of the novel bispecific FAP-DR5 antibody RO6874813 as a single agent or in combination therapy. An alternative approach to deplete CAFs is under development which uses a FAPα vaccine. Pre-clinical data demonstrated that FAPα vaccines were able to suppresses tumor growth and metastasis in colon and breast cancer models 198,199. However, no clinical trials have tested these vaccines. A summary of the ongoing clinical trials can be found in **Table 1**.

## Barriers to targeting CSCs

The primary barrier too many CSC targeting therapies has been toxicity. As CSCs share many markers and regulatory pathways with normal adult stem cells, many of these side effects are 'on target'. For example, notch inhibitors have significant gastrointestinal side effects related to targeting the gut stem cell niche; in clinical trials adverse gastrointestinal events affect up to 50% of patients 173,175. Thrombocytopenia, anemia, and neutropenia are common 172-175. Wnt inhibitors similarly show a number of gastrointestinal side effects and, related to Wnt signaling's role in bone remodeling, bone degradation 168,169. Importantly, the bone side effects could be treated with bisphosphonate therapy 168. Although Hedgehog pathway inhibitors have been commonly associated with a number of adverse events, these are generally of low grade, and management plans have been developed to alleviate these symptoms and limit discontinuation of treatment 200. The most concerning of these side effects are muscle spasms, which can become severe in some patients; however, a recent clinical trial (NCT01893892) demonstrated that levocarnitine could be used to partially alleviate these symptoms. Lastly, as noted above, trials targeting CD44v6 using bivatuzumab mertansine demonstrated serious skin toxicity 147,148. Whether this is drug or class specific remains unknown. However, other approaches, targeting CD44 using the broader-range anti-CD44 humanized antibody demonstrated significantly better tolerance at a >5x high dose than initial anti-CD44v6 trials and did not induced significant skin toxicity; instead, the dose limiting side effects were headaches and febrile neutropenia 144.

## Conclusion

Over the past decade, we have learned a significant amount about CSCs and begun to translate this into the clinic. Much like early cancer immunology studies, the earliest attempts at therapeutic approaches targeting CSCs have been disappointing. But the reality is that these attempts are still in their infancy. With increasing studies suggesting the branched nature of CSC hierarchies, it is likely that the best results will be obtained by targeting both arms of the CSC hierarchy. Furthermore, given the potential for dedifferentiation, agents such as chemotherapeutics or tyrosine kinase inhibitors, targeting bulk cells, will still be necessary. And much as better supportive care was needed to improve the tolerability of chemotherapy, supportive approaches for CSC-targeted therapy may be needed to overcome toxicity-related issues.

It is important to note that, even though we are still early in the course of CSC-targeting therapeutic development, we are beginning to see important clinical successes that increase hope that CSC targeting will indeed improve patient outcomes. Positive phase II trials with CSC targeting drugs metformin and disulfiram, showing significant improvements in patient overall survival, should encourage translational scientists to re-double their efforts at targeting CSCs clinically. Like the immune-oncology drugs, we believe that CSC-targeting drugs have an encouraging future.

## Figures and Tables

**Table 1:** Summary of select clinical trial results from the indicated CSC targeting drugs. PFS (Progression free survival), OS (overall survival), PR (partial response), CR (complete response), SD (stable disease), RR (response rate).

Drug Target	Drug	Clinical Trial	Cancer type	Highlights from Clinical Trials	Remarks	References
ALDH1	Disulfiram	Phase II (NCT003128 19)	Non-small lung cancer	Statistically significant Improvement in overall survival with some long-term survivors	Combination therapy with cisplatin and vinorelbine	http://theoncologist.alphame dpress.org/content/20/4/366. short
Unclear	Metformin	Randomized Phase II (NCT012109 11)	Pancreatic cancer	Metformin treatment did not improve PFS or OS.	Combination therapy with gemcitabine and erlotinib	https://www.ncbi.nlm.nih.go v/pubmed/26067687
		Randomized Phase II	Breast Cancer	Metformin treatment did not improve PFS or OS.	Combination with doxorubicin and cytoxan	https://www.ncbi.nlm.nih.go v/pubmed/30536182
		Randomized Phase II (NCT030717 05)	Lung Adenocarcin oma	Statistically significant improvement in PFS and OS (median OS 31.7 months vs. 17.5 months).	Combination with EGFR tyrosine Kinase inhibitor.	https://www.ncbi.nlm.nih.go v/pubmed/31486833
		Phase II (NCT015798 12)	Ovarian cancer	Statistically significant reduction in CSC. Median OS was 57.9 months.	Combination therapy with Carboplatin and Taxane	https://ascopubs.org/doi/abs /10.1200/JCO.2017.35.15_su ppl.5556
CD44	RG7356	Phase I (NCT013589 03)	Solid tumors	Modest single agent activity with 21% stable disease rate	Single agent therapy	https://www.ncbi.nlm.nih.go v/pmc/articles/PMC5346770/
CD133	CART-133	Phase I (NCT025413 70)	23 Patients with metastatic hepatocellul ar, pancreatic or colorectal carcinoma	3 Patients with PR, 14 SD and median PFS of 5 months.	Single agent therapy	https://www.ncbi.nlm.nih.go v/pmc/articles/PMC5993480/
FAK	VS-6063	Phase I (NCT025465 31)	Pancreatic ductal adenocarcin oma	Well tolerated but no clinical responses but 54% stable disease	Combination therapy with pembrolizumab and gemcitabine	https://ascopubs.org/doi/abs /10.1200/JCO.2018.36.4_sup pl.380
FAK	VS-6063	Randomized Phase II (NCT018706 09)	Pleural mesothelio ma	Merlin stratified pleural epithelioma. No PFS or OS improvement.	Single agent therapy vs. Placebo	https://www.ncbi.nlm.nih.go v/pubmed/?term=Maintenan ce+Defactinib+Versus+Placeb o+After+First- Line+Chemotherapy+in+Patie nts+With+Merlin- Stratified+Pleural+Mesothelio ma%3A+COMMAND- A+Double- Blind%2C+Randomized%2C+P hase+II+Study.
						
						
Wnt/β- catenin	Genistein	Phase I (NCT019857 63)	Colorectal cancer	Well tolerated, adverse events less than grade 4. Partial response in 61.5%	Combination therapy with FOLFOX and/or Bevacizumab	https://link.springer.com/arti cle/10.1007%2Fs00280-019- 03886-3
Wnt/β- catenin	Vantictumab	Phase I (NCT020053 15)	Pancreatic cancer	Partial response in 13 (41.9%) patient. Study terminated due to bone- related adverse events.	Combination therapy with nab-paclitaxel and gemcitabine	https://www.ncbi.nlm.nih.go v/pubmed/31338636
Notch	Gamma secretase Inhibitor	Phase II (NCT019857 63)	Pancreatic adeno- carcinoma	Stable disease was achieved in 25% of (12) and 6-month survival rate in 27.8% of patients.	Single agent therapy	https://www.ncbi.nlm.nih.go v/pubmed/24668033
		Phase I (NCT010983 44)	Pancreatic adeno- carcinoma	68% of patients had stable disease (stage IV pancreatic cancer) with a confirmed partial response in 5% of evaluated patients.	Combination therapy with Gemcitabine	https://www.nature.com/arti cles/bjc2017495
		Phase I (NCT016950 05)	Metastatic cancer	Anti-tumor activity observed in breast cancer, leiomyosarcoma and cystic carcinoma.	Single agent therapy	https://www.ncbi.nlm.nih.go v/pubmed/30060061
Hedgehog	Glasdegib	Phase II (NCT015460 38)	Acute Myeloid Leukemia	46.4% of 69 patients achieved CR. Median duration to CR is 94 days. Median OS is 14.9 months	Combination therapy with cytarabine and daunorubicin	https://onlinelibrary.wiley.co m/doi/full/10.1002/ajh.25238
IL-6	Stat 3 inhibitor OPB51602	Phase I (NCT011848 07)	Non-small lung cancer	PR observed primarily in patients with EGFR mutant lung cancer.	Single agent therapy	https://www.ncbi.nlm.nih.go v/pubmed/25609248 https://ascopubs.org/doi/abs /10.1200/jco.2014.32.15_sup pl.8028
IL-8	BMS-980253 Anti-IL8 Antibody	Phase I (NCT 02536469)	Solid tumors	73% stable disease rate with median treatment duration of 25 weeks	Single agent therapy	https://jitc.biomedcentral.co m/articles/10.1186/s40425- 019-0706-x
IL-8	Reparixin	Phase I (NCT023702 38)	HER2 negative Breast cancer	Well tolerated with a 30% response rate	Combined with Paclitaxel	https://www.ncbi.nlm.nih.go v/pubmed/28539464
FAPα	RO6874813 FAP-DR5 bispecific antibody	Phase-I (NCT025581 40)	Solid Tumors	Well tolerated 21% disease control rate (1 PR 6 SD).	Single agent therapy	https://mct.aacrjournals.org/ content/17/1_Supplement/A 092
